# *“I Do Feel Like a Scientist at Times”*: A Qualitative Study of the Acceptability of Molecular Point-Of-Care Testing for Chlamydia and Gonorrhoea to Primary Care Professionals in a Remote High STI Burden Setting

**DOI:** 10.1371/journal.pone.0145993

**Published:** 2015-12-29

**Authors:** Lisa Natoli, Rebecca J. Guy, Mark Shephard, Louise Causer, Steven G. Badman, Belinda Hengel, Annie Tangey, James Ward, Tony Coburn, David Anderson, John Kaldor, Lisa Maher, TTANGO Investigators

**Affiliations:** 1 The Kirby Institute, UNSW Australia, Sydney, NSW, Australia; 2 The Burnet Institute, Melbourne, VIC, Australia; 3 Flinders University International Centre for Point of-Care Testing, Flinders University, Adelaide, SA, Australia; 4 Apunipima Cape York Health Council, Cairns, QLD, Australia; 5 Ngaanyatjarra Health Service, Alice Springs, NT, Australia; 6 South Australian Health and Medical Research Institute, Adelaide, SA, Australia; 7 Queensland Aboriginal and Islander Health Council, Brisbane, QLD, Australia; Food and Drug Administration, UNITED STATES

## Abstract

**Background:**

Point-of-care tests for chlamydia (CT) and gonorrhoea (NG) could increase the uptake and timeliness of testing and treatment, contribute to improved disease control and reduce reproductive morbidity. The GeneXpert (Xpert CT/NG assay), suited to use at the point-of-care, is being used in the TTANGO randomised controlled trial (RCT) in 12 remote Australian health services with a high burden of sexually transmissible infections (STIs). This represents the first ever routine use of a molecular point-of-care diagnostic for STIs in primary care. The purpose of this study was to explore the acceptability of the GeneXpert to primary care staff in remote Australia.

**Methods:**

In-depth qualitative interviews were conducted with 16 staff (registered or enrolled nurses and Aboriginal Health Workers/Practitioners) trained and experienced with GeneXpert testing. Interviews were digitally-recorded and transcribed verbatim prior to content analysis.

**Results:**

Most participants displayed positive attitudes, indicating the test was both easy to use and useful in their clinical context. Participants indicated that point-of-care testing had improved management of STIs, resulting in more timely and targeted treatment, earlier commencement of partner notification, and reduced follow up efforts associated with client recall. Staff expressed confidence in point-of-care test results and treating patients on this basis, and reported greater job satisfaction. While point-of-care testing did not negatively impact on client flow, several found the manual documentation processes time consuming, suggesting that improved electronic connectivity and test result transfer between the GeneXpert and patient management systems could overcome this. Managing positive test results in a shorter time frame was challenging for some but most found it satisfying to complete episodes of care more quickly.

**Conclusions:**

In the context of a RCT, health professionals working in remote primary care in Australia found the GeneXpert highly acceptable. These findings have implications for use in other primary care settings around the world.

## Background

Point-of-care programs for sexually transmissible infections (STIs) have focused mainly on HIV and syphilis, due to funding priorities, and also the lack of accurate tests available for other STIs such as *Chlamydia trachomatis* (CT) and *Neisseria gonorrhoeae* (NG) which are easily curable with single-dose antibiotics. However with the recent availability of the first rapid, accurate, molecular diagnostic system (GeneXpert CT/NG assay) there is wider scope for the use of CT and NG point-of-care tests.[1,2] The GeneXpert system is also portable, in the sense that it can be packed and moved in a suitable case on wheels by one person, which adds to its utility.

The GeneXpert CT/NG assay is being utilised in the TTANGO (Test, Treat, ANd GO) trial.[3] TTANGO is a cross-over cluster randomised controlled trial underway in 12 remote Australian primary health care services in communities with a predominantly Aboriginal and Torres Strait Islander (hereafter referred to as Aboriginal) population. To our knowledge the trial represents the first use of a point-of-care molecular diagnostic test for STIs in a primary health care setting anywhere in the world.[3] GeneXpert platforms have been used for other infectious diseases, such as tuberculosis, but largely in laboratory settings.[4]

Provision of accurate testing and timely treatment through primary care services is a key STI control and prevention strategy. Point-of-care tests for CT and NG have the potential to increase the uptake and timeliness of testing and treatment and reduce the average duration of infectiousness,[5] thereby contributing to improved STI control in remote Australia and similar settings elsewhere. As utilisation of these new molecular point-of-care tests for CT/NG is a substantial change to clinical practice in these remote areas, and generally across the world, the TTANGO trial included comprehensive evaluation of the clinical, operational and cost-effectiveness of CT/NG point-of-care testing as well as its acceptability to health care staff and patients.

It is well established that the integration of point-of-care testing into STI clinical services in remote settings and elsewhere is not straightforward,[6] and that the mere availability of a point-of-care test does not ensure uptake by service providers or end users.[7,8] Acceptability of point-of-care testing to health care workers is necessary to support the introduction and sustainability of point-of-care testing, yet there has been a relative scarcity of qualitative research in this area.[9]

Acceptability of point-of-care test utilisation by health professionals is influenced by factors including: ease of use, in particular the number of manual steps involved and whether these are timed; reading and interpretation of results, especially if there is an element of subjectivity; specimen collection procedures and how complex or invasive they are; performance characteristics of the test (high sensitivity and specificity) and confidence in this; the extent to which point-of-care testing interrupts clinic workflow; and, the time taken to produce a result.[10–15] In the context of the TTANGO trial, we report on the acceptability of the GeneXpert CT/NG to primary health care staff working in remote Australian health services.

## Methods

### Ethics, consent and permissions

Ethical approval for the study was received from the West Australian Aboriginal Health Information and Ethics Committee, the West Australian Community Health Board Research Ethics Committee, the Townsville and Cairns Health Service District Human Research Ethics Committees and, the Aboriginal Health Research Ethics Committee of South Australia. Written informed consent was obtained from all participants. The trial is registered with the Australian and New Zealand Clinical Trials Registry (ACTRN12613000808741).

### Setting

Health care in remote Aboriginal communities is mainly provided through primary health care services, which are staffed by nurses and Aboriginal Health Workers/Practitioners mainly, with most having ‘fly in’ and ‘fly out’ Medical Officer support. In many remote communities in Australia, CT positivity and NG positivity are both around 20%.[16] Health services in these communities follow guidelines which recommend presumptive treatment in patients with STI symptoms or for patients living in communities with high rates of STIs and considered at high risk.[17–19] Patients who are asymptomatic for CT and NG (about 80% of those infected)[20] are treated on the basis of a laboratory test conducted at urban laboratories. Large distances to these laboratories and difficulties recalling patients [21–26] may result in one in six patients with an STI not being treated and delays in treatment of three weeks on average.[27]

### The TTANGO trial

TTANGO is a cross-over cluster randomised trial nearing completion in 12 Aboriginal community controlled health services in remote communities, mostly in Western Australia. TTANGO is measuring the impact of STI point-of-care testing on time to treatment and repeat infections, as well as its acceptability to clinicians and their patients. Participating health services were randomly assigned to either point-of-care or routine laboratory testing for CT and NG for one year before crossing to the opposite modality for another year. Nurses and Aboriginal Health Practitioners/Workers received on-site theory and hands on training of 2–3 hours duration, covering STI epidemiology, clinical management, and use of the GeneXpert and quality management practices. TTANGO Coordinators provided ongoing support via telephone, email and remote login access, with periodic visits (five over two years) offering face to face support and the opportunity to discuss STI testing data as a means of continuous quality improvement.[3]

### Sampling and participants

Interviews were open to all staff in participating health services that were trained and had approximately one year’s experience with GeneXpert testing. However, participation was influenced by clinic workload and other commitments on the day of data collection, and by the fly-in-fly-out nature of staffing (some staff were on leave at the time of data collection). Staff willing but unable to take part in a face to face interview were invited to participate in a telephone interview (resulting in three telephone interviews and 13 face to face). Recruitment of interview participants continued until the data were saturated and no new themes emerged.[28]

### Interviews

An interview guide was developed to explore staff experiences using the GeneXpert point-of-care test. Informed by a review of the literature, the guide included questions about a range of factors recognised to influence acceptability of point-of-care testing. The guide covered: the extent of participants’ involvement with GeneXpert testing; views on specimen collection, preparation, the testing process and accuracy of the test; the impact of point-of-care testing on traditional STI management practices and client flow; any particular ‘likes’ and ‘dislikes’ about the point-of-care device or the process/procedure; and associated attitudes about point-of-care testing. In addition, participants were asked if they could share any positive or negative anecdotes about testing patients. Interviews were conducted by the first author (who was also the project coordinator) and took 30–45 minutes.

### Data management and analysis

Interviews were digitally-recorded and transcribed verbatim. Transcripts were checked for accuracy against the recordings and to ensure familiarisation prior to analysis. Transcripts were uploaded into QSR Nvivo (Version 10), a qualitative data management and analysis program (QRS International PTY Ltd, Melbourne, Australia). Each transcript was systematically coded by the first author, who performed content analysis to examine frequencies of recurring codes and allocate salient themes.[29]

## Results

### Participants

A total of 16 participants were interviewed. The majority of participants were registered or enrolled nurses (75%), 56% were female, 37% identified as Aboriginal and the average age was 44 years (range 28–59 years). Most had performed ≤50 GeneXpert tests. (Table 1)

**Table 1:** Participant characteristics.

Participant variable	n (%)
**Australian jurisdiction where employed**	
Queensland	2 (12.5%)
Western Australia	11 (68.7%)
South Australia	3 (18.8%)
**Professional category**	
Registered or enrolled nurse	12 (75.0%)
Aboriginal health worker/practitioner	4 (25.0%)
**Age range (years)**	
26–35	3 (18.8%)
36–45	6 (37.5%)
46+	7 (43.8%)
**Sex**	
Male	7 (43.8%)
Female	9 (56.2%)
**Aboriginal or Torres Strait Islander**	
Yes	6 (37.5%)
No	10 (62.5%)
**Duration working in remote sector (years)**	
1–5	7 (43.8%)
6–10	2 (12.5%)
>10	7 (43.8%)
**Approximate number of GeneXpert tests performed since trial started**	
≤50	10 (62.5%)
>50	6 (37.5%)

### Overview of themes

**Fig 1:**
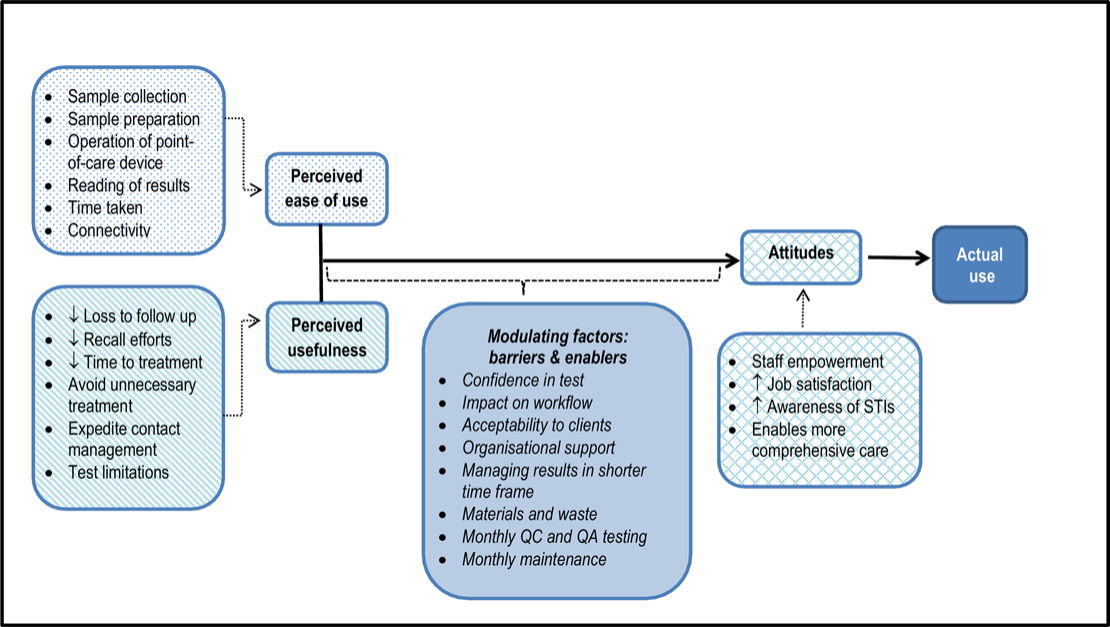
Influences on acceptability of Xpert CT/NG to operators in remote health services.

Adapted with permission. Original Copyright 1989 INFORMS. Fred D. Davis, Richard P. Bagozzi, Paul R. Warshaw (1989) User Acceptance of Computer Technology: A Comparison of Two Theoretical Models. *Management Science* 35(8):982–1003, the Institute for Operations Research and the Management Sciences, 5521 Research Park Drive, Suite 200, Catonsville, Maryland 21228, USA.

The data revealed a number of themes influencing the acceptability of the GeneXpert test including ‘Attitudes’, ‘Usefulness/utility’, ‘Ease of use’ and ‘Mediating factors: barriers and enablers’ (Fig 1).

Presenting the findings in this way represents an adaptation of the Technology Acceptance Model (TAM), which has previously been used to conceptualise technology acceptance. The model evolved from the information technology sector, but has also been applied in the area of health care.[30–32]

### Attitudes

#### Staff empowerment

Having access to a point-of-care device empowered staff, as they felt they were responsible for new technology which could improve the health of people in the community. This was particularly true for Aboriginal Health Practitioners/Workers.

*Getting my hands on that and knowing how to use that machine*… *I felt responsible yeah*, *made me feel a … part of it more … Like I’m not just a Aboriginal Health Worker*, *I was … helping and benefiting their health … [I]t’s exciting*, *and that’s what I tell a lot of the clients you know … I do feel like a scientist at times yeah … Makes you feel smart*! (Participant #9).

#### Job satisfaction

Given the delays associated with receiving results from laboratories in remote communities, and the challenges in locating clients for treatment when positive results are returned, participants commonly reported high levels of satisfaction with being able to test and treat on the same day.

*It most definitely had a big impact on my job … just the efficiency of testing someone there and then*, *treating them there and then … just cuts the* [work] *down a hell of a lot* (Participant #8).

Several also reported satisfaction from being able to alleviate client suffering and take a more proactive role in preventing transmission through prompt identification and treatment of existing cases.

*[W]e can interrupt that transmission of a positive case much more quickly … [I]n the past we had to wait three or four days and that person … can … see three or four other people within that three or four days before the test comes back* (Participant #8).

#### Increased awareness of STIs

Approximately half of the participants commented that the presence of the point-of-care device led to heightened awareness of STIs and testing, but were unsure if this would be sustained.

*It’s definitely made me think about STIs more*. *And because it’s been here for a year now*, *I’ve got more into the routine of doing a bit more of that*, *so I’ll probably still be doing more*, *but I think it will drop off—probably the amount of tests we’re doing overall anyway* (Participant #6).

Some participants felt that having the GeneXpert in place also raised awareness of STIs among community members. However views about this were mixed.

*There’s other people that have brought … their cousins … in because they’ve heard of the machine… and we still explain to them*, *you know*, *give them the whole education on the condoms and everything … not to go crazy just because we’ve got a machine that can pick it up in 90 minutes for ya [laughing]* (Participant #7).

*[E]ven though we’ve said just about every time that ‘we can test you on this machine and it will only take an hour and a half’*, *I don’t know if it’s actually got out there in the community*. *Because sexual health is already taboo … The machine isn’t known in the community*, *because people just don’t talk about this stuff* (Participant #1).

#### Enables more comprehensive care

Some staff commented on how the point-of-care test approach (and the 90 minute wait for results) created the space for broader health education and also gave an opportunity to invite clients to take part in a complete adult health check [an Australian government initiative that targets Aboriginal people, 15–54 years, to facilitate early detection, diagnosis and management of common, treatable conditions].

*[W]e had that 90 minutes to keep them around*, *so you’d use that opportunity to not just talk about sexual health but all kinds of health issues* (Participant #5).

*I can say … ‘You need an STI check*, *you want that today*, *it’s going to take an hour and a half for the result*, *you know what? … why don’t we knock out this ‘715’ … health check?’ And it gives that opportunity*, *and honestly like 70% of the time people will say ‘Oh well I’m gonna wait so let’s do that’ … and this gives us the opportunity to ask* (Participant #1).

### Perceived usefulness

#### Reducing loss to follow up

Many of the participants spoke of the significant population mobility in remote communities, and how delays associated with traditional laboratory testing meant that clients with positive results could not always be located for treatment. Participants felt that the point-of-care approach overcame this challenge.

*[I]f you can test and treat on the same day … it’s so much easier*, *cos* [because] *the population is so mobile … and between … football and funerals … and then there’s just the general moving between communities because of family*. *And you know people go off to shopping in Alice* [Springs] *or wherever*, *so generally if they’re in town on the day that you test they’re gonna be in town to treat them that day*, *but by the next day not necessarily* (Participant #12).

#### Reducing recall efforts

Delays in result turn-around associated with routine laboratory testing mean that clients with positive results need to be recalled to the clinic for treatment. Recall processes vary but are inevitably time consuming. Point-of-care testing virtually eliminates the need for client recall associated with routine laboratory testing, where clients are followed up days/weeks after specimen collection when laboratory results become available.

*It’s so hard and so time consuming to chase someone up that wants to be found*, *let alone doesn’t want to be found … [Y]ou need to go and pick them up … bring them in … bring them back home…[T]he amount of work and time that goes into picking up and bringing a patient in to* [name of service removed] *is extraordinary … So to have … a patient being treated in an hour and a half*, *cuts out 1000’s and 1000’s of dollars*, *I’m not kidding you*, *of manpower* (Participant #1).

#### Reducing time to treatment

For many participants, one of the overriding advantages of point-of-care testing was reducing the time between specimen collection and treatment provision. This was seen as particularly important in the context of high client mobility in many remote communities.

*[T]he woman was just about to jump in a car to go a funeral in another community*, *and she said ‘Yep*, *I’ll come with you straight away’ … we went to the clinic*, *I treated her*, *we had a chat*, *I didn’t even have a chance to take the bloods because they were knocking on the door ready to go to the funeral*. *Now had we not had that turn around in result*, *that woman would have been gone from our community for perhaps up to a week … until she got back to us she wouldn’t have got treated* (Participant #12).

Several participants highlighted the additional benefit of reducing the time to treatment for pregnant women, whose pregnancies might otherwise be at risk of the complications of STIs.

#### Avoiding unnecessary treatment

One participant shared the story of a symptomatic client where the clinician chose to deviate from recommended guidelines and wait for the point-of-care result rather than treat on the basis of symptoms. The point-of-care result was negative, and subsequently the client was treated for a urinary tract infection rather than an STI.

*I had a lady that came who was concerned that she had an STI and tested her and came back negative and so we treated her for a urine infection rather than a STI which is really good* (Participant #10).

#### Expediting contact tracing

Some participants commented that the point-of-care approach enabled the partner notification process to commence more quickly.

*[A] guy came in and he got the test … tested positive*, *that was sort of in the morning*, *by the afternoon we had that same client’s partner back in the clinic here … we had them both treated* (Participant #8).

#### Test limitations

Some of the participants commented it was disappointing that POC testing for trichomonas was not available, as it is highly prevalent in many remote communities. Several also noted that routine reference laboratory test reports often include antibiotic sensitivities, and that this information is not available via the point-of-care device (at this time) and would not be available if samples were no longer sent to the laboratory.

*[I]t would also be good if it had trich* [trichomonas] *… that’s another thing*, *it doesn’t determine whether its amoxicillin resistant or not* (Participant #14).

### Perceived ease of use

#### Sample collection

Respondents liked the fact that the point-of-care device could process both urine and swab specimens (the specimen type collected varied by site according to local guidelines). One participant (from a health service where urine rather than swab specimens are routinely collected from women) indicated that clients found the collection process less invasive and more acceptable than that associated with swabs.

*I just found the urine was less invasive*, *people were more compliant*, *people were more willing to do a urine specimen than do a swab … so I just used the urine* (Participant #5).

#### Sample preparation

Many participants reported that it was easier to prepare a urine sample than a swab sample, although this tended to change over time with increasing experience.

*Perhaps the urines were easier*, *a little less fiddly maybe*, *but I did so many swabs that it didn’t make any difference to me … It was easy*, *I think it took a few goes to get it correct*, *but then it was like you just ‘had it’* (Participant #12).

Participants had mixed views on using the disposable pipette, which was central to sample preparation. Some found it intuitive, but those who didn’t commonly modified practice to use a 1mL syringe rather that the pipette provided with the test kit.

*It didn’t bother me- we’re pipette people anyway*. *We have to use pipettes for βHCGs*, *I use sterile pipettes when I do a UA* [urinalysis] *so it made no diff [*difference] *to me* (Participant #15).

*I didn’t use the pipette*, *I found it difficult*, *so I just used the 1mL syringe which I found easier to hold*, *easier to draw it up and more accurate* (Participant #5).

A few participants commented that operators need to be very disciplined with aseptic technique during the sample preparation process, and that this could be time consuming.

#### Operation of point-of-care device

Most participants found the GeneXpert easy to use and reliable.

*I’m not the most computer literate* [laughing] *… There were only a couple of screens you had to go to*, *the machine opened every time*, *you know ‘start the test’*, *look for the result- bang*. *It was simple*, *I don’t think you could make it much more simple*, *very user friendly* (Participant #12).

However, several reported challenges in initially understanding which information was required to be entered in each field on the ‘start a test’ template on the computer- in particular the sample ID field. Some found the data entry process time consuming and suggested that this would be greatly simplified if the machine was linked to the patient management system and these data fields could self-populate.

*Yeah*, *I wasn’t very happy with that*, *I found that quite difficult sometimes*, *and the confusion with*, *it had* [a] *UR number and then another number… I just felt it was ‘busy’ or confusing sometimes*, *I’m not a great computer person* (Participant #15).

*[T]he identification of the patient and the rest of the data that you’re supposed to* [enter]*–it’s more convenient if it’s in the computer already* (Participant #11).

Several commented about the noise (beep and whirr) of the machine; this was raised more often by staff whose office space was co-located in a clinic/procedure room. Others also expressed frustration with the need to upgrade software periodically- which happened in the early stages of the CT/NG assay release.

#### Reading results

Participants liked the fact that there was no element of subjectivity in interpreting test results, which are displayed as either ‘detected’ or ‘not detected’ and with result text highlighted in corresponding red or green.

*[It’s] pretty clear*. *Seeing the green and the red light there* (Participant #11).

#### Time taken

Most participants indicated that the time required to perform and generate a test result was acceptable, given the many benefits of testing at the point-of-care.

*[I]t was a pretty simple process*, *… it was ‘do the test*, *get the result*, *… make sure the paperwork’s done and that’*, *I mean honestly it was hardly any extra work … it just depends on your point of view … I thought it was an advantage to us and I didn’t think it was much extra work* (Participant #12).

#### Connectivity

Several participants commented that the process of documentation (transcribing test results from the GeneXpert into patient notes) was time consuming. This seemed to be exacerbated if the point-of-care device and the computer being used to access patient files were located in a different room.

### Modulating factors: barriers and enablers

#### Confidence in test

Participants generally expressed high levels of confidence in point-of-care test results and were happy to treat patients on this basis.

*Yeah 100% confident*, *because even though we were doing the TTANGO test* [trial], *we still had to send our specimens to pathology and they were 100% spot on* (Participant #3).

*[T]he test results seemed to be pretty much completely accurate with what we got back from pathology*… *I was pretty confident … I think there were only one or two discrepancies… but if there was a positive result for chlamydia or gono I felt that I could treat it quite easily* (Participant #6).

However, a few participants expressed concern about the potential for false positive results, based on a perceived risk of cross contamination from surrounding surfaces and circulating dust during sample preparation.

*[B]ecause of the sensitivity of it and the way you can cross contaminate the specimens very easily … because there’s a lot of dust in the clinics … It’s very easy to contaminate your specimens*, *if you’re not diligent* (Participant #14).

Confidence in the test appeared to be influenced by the frequency of experience with invalid results and perceived concerns (unfounded) related to the impact of power failures on the machine.

*I was more confident in the urine results … I had a lot more … invalid results with the swabs*, *and I don’t know why … they watched my technique and there was nothing wrong with my technique* (Participant #15).

*[W]as the machine properly calibrated at all times … and if there was any like power failures or anything like that in the time that we were using it*… *because we’ve had a lot of interruptions with power*, *through like power works in the area and plus storms and cyclones and switching the machines off … making sure its re-programmed up properly* (Participant #4).

#### Impact on workflow

At many health services, clients often left the clinic after the consultation while waiting for the results of the point-of-care test, with staff arranging to call them later in the day with results. At some health services clients were still in the clinic at the time results became available. Either way participants generally reported that the point-of-care testing was minimally disruptive. The exception to this was feedback from staff in very busy/understaffed services, who felt that the point-of-care approach created ‘another thing’ to remember and track in an already busy environment.

#### Acceptability to clients

When reflecting on their clients’ experience of point-of-care testing, more than half of the participants reported a high degree of acceptability. Participants thought that their clients were generally pleased to receive their test results more quickly.

*[I]f it comes back negative then it’s peace of mind for them*, *but if it comes back positive they know they’re going to get treated straight away*, *I think either way it’s a positive … but from what I’ve seen people are really happy to get a faster result and get treated faster- or not*, *if it’s a negative* (Participant #3).

#### Organisational support

Organisational and management support of staff involved in point-of-care testing and STI work in general may have influenced acceptability for some participants.

*I had a big break away from the machine because I had time away and I got moved around in positions … and … all of a sudden it was all behind*. *If someone’s not driving it*, *um*, *tests slow down*, *people forget … Someone should drive it in house*… *I mean someone who does STIs full time … but unfortunately the STI coordinator here … doesn’t even have a day*, *one day any more to take care of STIs … That’s not cool*, *you need someone* (Participant #1).

#### Managing results in shorter time frame

Participants were asked whether they felt that managing STI test results in a shorter time frame was more challenging for them. Responses were mixed. Some commented that the immediacy of the result made the testing experience more confronting for them and their clients.

*You get a test that’s positive and usually it’s the partner finding out that the other partner must be doing the sly on them because they’ve got an STI and they haven’t been with anyone for years except for their partner … [W]ithin an hour and a half its* [the result] *very fresh … [I]t does start sooner which opens up a whole other field … because when you’ve got a person sitting there and they find out they’ve got an STI*, *particularly if they’re in a long term relationship … sometimes I really worry about the next couple of days of you know the partner coming in* [having been] *beat up or*, *which happens a lot here* (Participant #1).

Other participants felt that it made it easier to discuss the result with the client, at a time when the testing was fresh in their mind. Respondents also indicated that actioning results is simpler in a shorter time frame, as patients can often be found more easily, treatment can be provided and the episode of care completed on the day of testing.

#### Materials and waste

One participant, admittedly from a very small clinic, remarked about the space and storage (air conditioned) requirements for test kits.

*[S]torage was a bit of an issue at the start … because we had to figure out where they were going to be refrigerated and all that* (Participant #13).

This same participant was worried about the environmental impact of testing, commenting on the numerous layers of packaging that surrounds the specimen collection kits. This participant also mentioned the yellow infectious waste bins that are required to enable safe disposal of used test cartridges (which must be destroyed in an enclosed incinerator). These bins then need to be trucked with other infectious waste to a suitable disposal facility.

#### Monthly quality control (QC) and quality assurance (QA) testing

The need for staff to perform quality control testing once per month and to test a panel of four external quality assurance swabs twice per year was seen by some as an important task to ensure the point-of-care test was performing well, rather than a burden. However some admitted to forgetting to do QC testing every month.

*That’s always brilliant because it’s good to know that the machine is actually working and giving like correct results … Makes it feel like you know a little bit better … that it is reading ok …We probably could have done more*, *but … you get side tracked* (Participant #10).

#### Monthly maintenance

Very few participants commented on the small amount of monthly module maintenance (approximately 10 minutes of work). Where comment was made this did not appear to raise concern.

*That was easy*, *that was fine*, *it doesn’t take long*, *you just have to clean those rod things* (Participant #6).

## Discussion

Our findings indicate a high level of acceptability of the GeneXpert CT/NG among health professionals working in remote primary care in Australia where there is a high burden of STIs and significant distances from laboratories.

Most staff displayed positive attitudes towards the point-of-care device, and indicated that it was both easy to use and useful in their clinical context. They indicated that point-of-care testing had improved STI management, resulting in more timely and more targeted treatment, earlier commencement of partner notification, and reduced follow up efforts associated with client recall. Staff expressed confidence in point-of-care test results and treating patients on this basis, and reported greater job satisfaction—feeling more in control of STI testing and patient health, particularly Aboriginal staff. While point-of-care testing did not negatively impact on client flow, several found the required manual documentation processes time consuming, and felt that improved connectivity and automatic transfer of results between the GeneXpert and the patient management system could facilitate better workflow. Managing positive test results in a shorter time frame was challenging for some but most participants found it more satisfying being able to complete the episode of care on the same day.

The GeneXpert has largely been used in hospital settings for TB testing and more recently for other purposes,[33] [34,35]and deployed for TB testing in primary and remote health care settings.[36] While there is an increasing body of literature on test performance and clinical impact related to these applications, there are few studies that refer to acceptability [33,37] and none to our knowledge that formally use qualitative methods to explore acceptability or that were conducted in primary care settings. Goldenberg and colleagues explored acceptability and ease of use (using a short questionnaire) of GeneXpert testing for Clostridium Difficile in a hospital setting; most staff found the device easy to use, perceived point-of-care testing to be an acceptable part of their role, liked being able to perform testing themselves, agreed that results were available more quickly than the laboratory based test and that rapid result turn- around facilitated better management of patient beds.[33] The interview findings described here broadly align with those described by Goldenberg and colleagues, however due to the qualitative nature of our interviews, we provide more information about the reasons for high acceptability and the various factors that impact on this. Our findings also demonstrate for the first time that this technology is acceptable to staff working in small, remote health services, and that staff acceptability should be an enabler to implementation and uptake in this sector. Our findings are also consistent with the Technology Acceptance Model [30–32] and provide a case study of its application to new technology in remote primary health care settings.

Our data identified three strong acceptability themes. Consistent with other studies participants generally reported that the point-of-care test system was user-friendly, reliable, and resulted in benefits for them (reducing recall efforts) and for their patients (reducing time to treatment and reducing unnecessary treatment) without disrupting workflow.[10–15] When the study commenced, some stakeholders raised concerns about the 90-minute wait for results, identified as an important determinant of staff acceptability in previous research, with 20–30 minutes considered by frontline health professionals as acceptable for an STI test.[12,14,15] However the 90-minute wait was not raised as an issue by our participants, possibly because most clients opted to leave the clinic and return in the event of a positive result, so staff workflow was not negatively impacted. Participants may also have felt that this timeframe was acceptable and highly favourable when compared to the time taken to receive routine laboratory results in most remote settings.

Our finding of high acceptability and, in particular, benefits to staff and patients, needs to be interpreted in the context of sexual health care service delivery in remote Aboriginal communities. In most urban sexual health services, patients are sent their results by text message in a few days. However in remote Aboriginal communities, Aboriginal health practitioners/workers and nurses, who are often responsible for locating patients, are part of and known to the community, so they need to be careful and sensitive about how they seek out individuals, which in turn means recall can be very time consuming. Also mobility is high in remote Aboriginal communities, due to personal, family or cultural reasons, and additional efforts may be needed to locate patients, including contacting other health services to see if they can assist.[38] These processes in remote Aboriginal communities are similar to systems in countries in the Asia-Pacific region where village health workers or volunteers are tasked with locating community members for a range of health promotion interventions.[39,40]

There were also some minor points of dissatisfaction raised by staff interviewed. The lack of automated data flow between the GeneXpert and the patient management system and other concerns relating to connectivity will be addressed before wider implementation of the GeneXpert system in Australia. In addition, it will be important to work with implementing staff to ensure greater clarity around patient and sample identifiers used in the GeneXpert device; this issue could readily be resolved by modifying the software data fields and including additional resource material on this subject in future training materials.

A few staff had concerns about the risk of cross contamination between samples, however the closed cartridge system and the very low occurrence of discrepant results (specimens tested on the GeneXpert were also tested at reference laboratories for monitoring purposes during TTANGO) suggest that this is unlikely; it is possible that during training we over-emphasised the need to take care with specimen processing to reduce any risk of false positive results, but similarly, the attention to detail in training may be part of the reason that cross contamination of samples has not been an issue.

One participant noted that the GeneXpert CT/NG test result does not report on NG antibiotic sensitivity, which is otherwise conducted by Australian laboratories using cultured isolates (with one laboratory also using molecular methods to test for penicillinase producing N. gonorrhoea on all positive NG tests).[41] This issue has been raised by stakeholders in previous research [42] and it is critical that in the future point-of-care NG test processes support specimens to be available for NG antimicrobial resistance surveillance.

While our findings suggest that ideally one or two staff members should be allocated responsibility to ‘drive’ point-of-care testing within a service, this may be difficult to sustain given the realities of turnover of staffing in this context of remote Australia. Working in remote primary care is recognised to be extraordinarily challenging.[43,44] Staff face competing demands, with chronic under-staffing and over-reliance on temporary staff to fill essential positions. Those in permanent positions or on longer term contracts are often required to back fill critical roles. High level organisational and management support is therefore critical if point-of-care testing for CT/NG is to be successfully integrated into this sector. It is, however, particularly encouraging that there was a high level of acceptability among Aboriginal health practitioners/workers. These staff are often the most stable members of the remote health workforce, but may perceive themselves to be low in the health workforce hierarchy and have a limited scope of practice. Findings suggested that having a role in GeneXpert testing empowered staff, giving them added responsibility for something very important, that could help improve the health of their community.

This is the first study to explore acceptability of the GeneXpert CT/NG in remote primary care in Australia or elsewhere. The main strength of this study is that we used qualitative methods, which are well suited to open-ended enquiry or exploratory research where little is known about the issue under study. Our study also has several limitations. The small sample size and non-random nature of the sampling strategy limit the generalisability of the results, however we did interview key staff involved in GeneXpert testing in seven of the 12 services. The interviewer was not completely independent of trial implementation; however there is no reason to believe that her relationship to the project affected responses. At this stage we are unable to comment in detail on whether there were acceptability issues beyond the users themselves.

## Conclusions

Use of the GeneXpert in primary care services is a substantial change to clinical practice. In the context of a RCT, health professionals working in remote primary care in Australia found the GeneXpert CT/NG highly acceptable. These findings have implications for use in other primary care settings around the world. The next phase will involve a long-term program of GeneXpert CT/NG testing and an evaluation of sustainability.
